# Genome Sequence of Mycobacteriophage Mindy

**DOI:** 10.1128/genomeA.00596-15

**Published:** 2015-06-18

**Authors:** Welkin H. Pope, Nicholas I. Bernstein, Christina S. Fasolas, Nadia Mezghani, Catherine A. Pressimone, Priyanga Selvakumar, Ann-Catherine J. Stanton, Jonathan S. Lapin, Ashley K. Prout, Sarah R. Grubb, Marcie H. Warner, Charles A. Bowman, Daniel A. Russell, Graham F. Hatfull

**Affiliations:** Department of Biological Sciences, University of Pittsburgh, Pittsburgh, Pennsylvania, USA

## Abstract

Mycobacteriophage Mindy is a newly isolated phage of *Mycobacterium smegmatis*, recovered from a soil sample in Pittsburgh, Pennsylvania, USA. Mindy has a genome length of 75,796 bp, encodes 147 predicted proteins and two tRNAs, and is closely related to mycobacteriophages in cluster E.

## GENOME ANNOUNCEMENT

Bacteriophages, the most abundant biological entities in the biosphere, are viruses that infect bacterial hosts (1). Phages genetically are highly diverse and have therapeutic potential for controlling bacterial pathogens (2). A large collection of mycobacteriophages—phages that infect mycobacterial hosts—offers insights into phage evolution and provides a toolbox for genetic manipulation of *Mycobacterium tuberculosis* (3). The Science Education Alliance Phage Hunters Advancing Genomics and Evolutionary Science (SEA-PHAGES) program involves large numbers of students at multiple institutions to gain research experience through phage isolation and genomic analysis (4).

Mycobacteriophage Mindy was isolated by direct plating of a soil sample from Pittsburgh, Pennsylvania, and DNA was isolated following plaque purification and phage amplification. The genome was sequenced on an Illumina MiSeq platform using140-bp single-end reads. Reads were assembled using Newbler into a single major contig with 4,365-fold coverage. The Mindy genome is 75,796 bp long with 9-bp 3′ single-stranded DNA extensions with the sequence 5′-CGCTTGTCA. The genome has a G+C content of 63.0%.

GeneMark and Glimmer were used to generate a preliminary autoannotation—using heuristic and *Mycobacterium smegmatis* models—which was then revised following manual inspection. A total of 147 predicted protein-coding genes were identified and two tRNA genes were identified using Aragorn and tRNAscan-SE. Gene functions were predicted using BLASTp, HHpred, and domain searches in the program Phamerator (5) and predicted functions were assigned to 43 of the 147 predicted genes.

Mycobacteriophage Mindy is closely related to phages Cjw1, 244, Kostya, SirDuracell, Porky, and Rakim and joins them in cluster E. The most closely related phage is Henry (6), with which it shares 99% nucleotide identity across a span of 98% of its genome. As in other cluster E phages, the genome architecture is such that there are two large blocks of rightward-transcribed genes and two smaller blocks of leftward-transcribed genes, with several interruptions of 1 to 2 genes. The terminase large subunit (gp9) contains an intein and the capsid subunit (gp13) is predicted to assemble similarly to the HK97 capsid (7). The Mindy genome includes two related adjacent genes *94* and *95* (51% amino acid identity) and may be a precursor to those phages containing homologues of only one of these (e.g., Henry *93* and Toto *93*). Two Mindy gene products are predicted to have interesting DNA binding properties: gp75, which is predicted to be structurally related to DNA processing protein A (8), and gp57, which is structurally related to the MfpA protein of *M. tuberculosis* (Rv3361c), which confers resistance to fluoroquinolones by mimicking the structure of dsDNA and binding to DNA gyrase (9). Mindy gp56 is predicted to be expressed during early lytic growth, and it is unclear whether it binds to the host MfpA protein or to a different target through DNA mimicry.

### Nucleotide sequence accession number.

The Mindy genome sequence is accessible from GenBank under the accession number KR080204.
